# On nonlinear viscoelastic deformations: a reappraisal of Fung's quasi-linear viscoelastic model

**DOI:** 10.1098/rspa.2014.0058

**Published:** 2014-06-08

**Authors:** Riccardo De Pascalis, I. David Abrahams, William J. Parnell

**Affiliations:** School of Mathematics, University of Manchester, Oxford Road, Manchester M13 9PL, UK

**Keywords:** viscoelastic, quasi-linear, Fung, strain energy function, hyperelastic, biological soft tissue

## Abstract

This paper offers a reappraisal of Fung's model for quasi-linear viscoelasticity. It is shown that a number of negative features exhibited in other works, commonly attributed to the Fung approach, are merely a consequence of the way it has been applied. The approach outlined herein is shown to yield improved behaviour and offers a straightforward scheme for solving a wide range of models. Results from the new model are contrasted with those in the literature for the case of uniaxial elongation of a bar: for an imposed stretch of an incompressible bar and for an imposed load. In the latter case, a numerical solution to a Volterra integral equation is required to obtain the results. This is achieved by a high-order discretization scheme. Finally, the stretch of a compressible viscoelastic bar is determined for two distinct materials: Horgan–Murphy and Gent.

## 1. Introduction

The study of nonlinear viscoelastic deformations of solid materials has a very long history, with a consequent proliferation of a diverse and extensive array of constitutive models. For a comprehensive overview of the topic, the reader is directed to the recent paper by Wineman [1] and related references therein. The subject is still active, with models continuing to be developed across the field, from highly mathematical approaches where implementation is not a concern to very applied studies where ease of application is essential. Each approach has its advantages: the models that more accurately describe the microphysics tend to prove difficult to employ in practical engineering or biomedical situations, whereas simpler approaches often miss crucial details. If the viscous model assumes a fading memory effect of the strain history, then this usually gives rise mathematically to Volterra-type integral equations, which in the linear case can generally be solved either analytically or numerically. However, in the nonlinear case (of interest to describe finite deformations), these problems are difficult to solve by any methods, even finite-element approaches. It is therefore crucial to develop constitutive models that are simple enough to be amenable to straightforward (and rapid) solution methods, yet include enough detail to capture the important physics underlying these relevant materials. One such ‘compromise’ approach was offered by Fung [2], in order to study the uniaxial elongation of biological soft tissue. His constitutive assumption, often called *quasi-linear viscoelasticity* (QLV) or *Fung's model* of viscoelasticity, assumes that the viscous relaxation rate is independent of the instantaneous local strain. This model, which is a special case of a more general Pipkin–Rogers constitutive model [3], predicts that at any time a stress that is equal to the instantaneous elastic stress response decreased by an amount depending on the past history, assuming that a Boltzmann superposition principle holds.

Fung's QLV model is perhaps the most widely used today, but has met with some criticism mainly because it has been suggested that it does not always yield physically ‘reasonable’ behaviour. Of course, as with any constitutive model for a complex nonlinear material, QLV has limitations. However, it can be expected to be appropriate for materials whose relaxation-rate coefficients are weakly dependent on deformation, or where the deformation comprises small perturbations about a large initial deformation. Various experimental results have appeared in the literature that appear to confirm that QLV is, in practice, a reasonable model for a range of materials; see for example [4–6].

The apparent deficiency of QLV will be discussed below, but the purpose of this paper is to reappraise Fung's approach, suggesting how it can be reformulated against existing interpretations in the literature. It will be seen that the form offered herein has similarities to other viscoelastic formulations, including that by Simo [7], and most importantly exhibits behaviour that makes it both (relatively) easy to use and physically useful.

Analysis will commence with the general tensorial form of (QLV) proposed by Fung [2]

1.1 $$\pi (\text{X},t)=\int_{-\infty }^{t}\text{G}(t-\tau )\frac{\partial \pi^{e}(\text{X},\tau )}{\partial \tau }d\tau ,$$

where **G**(*t*) is, in comparison with linear theory, the stress relaxation second-order tensor, **Π** denotes the second Piola–Kirchhoff stress, while **Π**^e^ is an instantaneous strain measure. The latter may be thought of as an equivalent (instantaneous) *elastic* stress, hence the superscript ‘e’. This tensorial integral identity is the natural generalization of the simple one-dimensional relationship proposed by Fung [2], which preserves *objectivity*.

As mentioned, thanks to the relative simplicity of Fung's approach over more general nonlinear viscoelastic models, it has proved extremely popular. This is especially true in the biomechanics field where it has been employed to predict the large deformations of soft tissues. However, despite the widespread use of QLV, published studies (especially in the incompressible limit) appear to interpret (1.1) in different ways, which then lead to quite different results. In the paragraphs below, a number of common approaches are discussed, where the interpretations of the model may be questioned. The main aim of this paper is then to re-derive QLV, starting from basic principles and also to ensure consistency in the limit of infinitesimal deformations, thus recovering the Boltzmann theory of linear viscoelasticity.

The simplest interpretation of Fung's relation is to assume a purely one-dimensional homogeneous deformation. This is attractive for its ease of use and has been employed extensively in biomedical applications [8–10], for example to model the shearing deformation of brain tissue [6]. However, in practical applications, especially for incompressible or near incompressible materials purely one-dimensional deformations, such as pure uniaxial extension, will not be realizable. Thus, a tensorial form of QLV must be employed. As mentioned, this relation must, at the minimum, satisfy objectivity, and hence the form chosen in (1.1). Nevertheless, a number of authors have erroneously expressed Fung's relation not for the second Piola–Kirchhoff stress, which guarantees objectivity, but in terms of other stress measures. For example, see Fung's original discussion on the subject in [2, page 253], in which the QLV relation is expressed in terms of the Kirchhoff stress! In the latest version [6.12] of ABAQUS, although the viscoelastic constitutive model is objective, the model is rather heuristic and as we shall see later, it assumes the same relaxation behaviour in both the shear and bulk parts of an isotropic material. Further technical aspects of objectivity are discussed in the paper by Liu [11].

Another approach to QLV sometimes employed in the literature (e.g. [12–14]) is, in an analogous fashion to nonlinear elasticity, to write the stress in terms of the instantaneous derivative (with respect to the principal stretch) of a strain energy function. Clearly, in this case the strain energy function must be dissipative and depend on the history of the strain, and so these authors express it as a fading-memory integral with integrand given as a hyperelastic strain energy function. This approach may be somewhat hard to justify and does not allow the user to consider the problem in terms of an auxiliary instantaneous measure of strain, i.e. the effective elastic stress term **Π**^e^ given above.

Perhaps, a more subtle point to those mentioned above is the choice of **Π**^e^ in (1.1). In this paper, the reasonable assertion is made that **Π**^e^ must be zero whenever the deformation is zero. However, this point is often not recognized, especially for incompressible materials, as can be seen, for example, by inspection of the integrands (setting the stretch equal to unity) in the articles by [15] (see eqns (8) and (12) therein), [16] (eqns (9) and (13)) and [17] (eqn (9)). Note that this requirement is satisfied in the one-dimensional models in [8,9] but not in [10]. If **Π**^e^ does not vanish for zero deformation then, in general, it can be expected that the actual stress field **Π** will be non-zero prior to the application of the imposed load or stretch, i.e. the solution will be non-causal! However, for incompressible materials, an arbitrary pressure term (Lagrange multiplier) can be adjusted so that the stress field is indeed causal, but then it must take a specific form at later times. That is, whenever the material is deformed, and then returns back to zero, the stress **Π** will instantaneously return to zero too. This is clearly an unrealistic consequence of the particular choice of model, for, as Fung states [2, page 228]: ‘the tensile stress at any time *t* is equal to the instantaneous [elastic] stress response … decreased by an amount depending on the past history’. This issue is examined further in §4*a*.

There are other variants of QLV employed in the literature that offer slightly modified forms of the governing equation to that suggested by Fung. The reader is referred, for example, to articles [18–20]. In this paper, the method employed follows closely that proposed by Fung but does not exhibit any of the limitations just discussed.

The paper is organized as follows. In the following section, the usual (Boltzmann) linear viscoelastic model is reviewed by the way of introduction to a new interpretation of Fung's QLV theory, presented in §3. In §4, results from the new model are contrasted with those in the literature for the case of uniaxial elongation of a bar: in §4*a* for an imposed stretch of an incompressible bar, in §4*b* for an imposed load, and in §4*c* for stretch of a compressible bar. A numerical solution to a Volterra integral equation is required for the results in §4*b*, and the discretization (time-stepping) procedure used is described in appendix A. Finally, concluding remarks are offered in §5.

## 2. Boltzmann's linear viscoelastic law

It is helpful to commence analysis by recapping the theory of linear viscoelasticity. Under the assumption of isotropy, infinitesimal elastic deformations can be described by the constitutive law

2.1 $$\sigma (t)=2\mu (\epsilon (t)-\frac{1}{3}\text{tr}(\epsilon (t))\text{I})+\kappa \text{tr}(\epsilon (t))\text{I},$$

where ***σ*** and ***ϵ*** are the second-order stress and strain tensors, respectively, and *μ* and *κ* represent the infinitesimal shear modulus and modulus of compression (or bulk modulus), respectively. To incorporate viscoelastic behaviour, the most natural extension of (2.1) is to assume that the stress *remembers* the past history of the rate of strain with some *fading memory*, and then apply the *superposition principle* (or Boltzmann's principle); hence,

2.2 $$\sigma (t)=2\int_{-\infty }^{t}\mu (t-s)\frac{\partial }{\partial s}\left(\epsilon (s)-\frac{1}{3}(\text{tr}\epsilon (s))\text{I}\right)ds+\int_{-\infty }^{t}\kappa (t-s)\frac{\partial }{\partial s}(\mathrm{tr}\epsilon (s))\text{I}\;ds,$$

where *μ*(*t*), *κ*(*t*) are now time-dependent *relaxation* functions, and the lower limit of the integral must be taken from −∞ in order to correctly consider the initial deformation. Integrating (2.2) by parts, and assuming that the deformation commences at *t*=0, yields

2.3 $$\begin{array}{rl} \sigma (t) & =2\mu (0)\left(\epsilon (t)-\frac{1}{3}(\mathrm{tr}\epsilon (t))I\right)+2\int_{0}^{t}\mu^{\prime }(t-s)\left(\epsilon (s)-\frac{1}{3}(\mathrm{tr}\epsilon (s))I\right)ds\\ & \;+\kappa (0)\mathrm{tr}\epsilon (t)I+\int_{0}^{t}\kappa^{\prime }(t-s)\mathrm{tr}\epsilon (s)I\;ds, \end{array}$$

where the ′ denotes differentiation with respect to the argument of the function. Note that this expression incorporates any jump discontinuity when the motion starts. Moreover, the first term in (2.2) (or equivalently the first two terms in (2.3)) is trace free, or deviatoric, and accounts for shear deformations and loss, while the second term accounts for the hydrostatic part representing compressive deformations and loss.

Now, in the elastic case (2.1), incompressibility may be considered as the dual limit of κ/μ→∞ and trϵ→0, which results in the stress–strain relationship

2.4 σ(t)=2μϵ(t)−p(t)I,

i.e. the second term in (2.1) yields a finite non-zero limit, where *p*(*t*) may be considered as a Lagrange multiplier of incompressibility. For the viscoelastic counterpart, the limit of incompressibility can be considered in a similar fashion, noting first that μ(∞) and κ(∞) are the long time shear and bulk moduli (the equivalent of *μ*,*κ* in the elastic case), respectively. Thus, taking κ(∞)→∞ (which implies κ(t)→∞ for all *t* owing to the fading memory assumption) and trϵ→0 in (2.2) (or in (2.3)), it is found that

2.5 $$\kappa (0)\mathrm{tr}\epsilon (t)+\int_{0}^{t}\kappa^{\prime }(t-s)\mathrm{tr}\epsilon (s)ds\to -p(t),$$

where the limit is again assumed to have a non-zero finite value, −*p*(*t*). Thus, in the limit of incompressibility, equations (2.2) and (2.3) become

2.6 $$\sigma (t)=-p(t)I+2\int_{-\infty }^{t}\mu (t-s)\frac{\partial }{\partial s}\epsilon (s)\;ds$$

and

2.7 $$\sigma (t)=-p(t)I+2\mu (0)\epsilon (t)+2\int_{0}^{t}\mu^{\prime }(t-s)\epsilon (s)\;ds,$$

respectively.

## 3. Quasi-linear viscoelasticity

When the strain is not infinitesimal, linear theory becomes inappropriate to describe deformations; hence, a nonlinear constitutive law has to be considered. As discussed in the Introduction, Fung's hypothesis (1.1) is examined here as a means of describing the motion of viscous nonlinearly elastic materials. In his renowned work [2], Fung introduced this quasi-linear constitutive model in order to capture the nonlinear stress–strain relationship of living tissues; however, it also has applicability to elastomeric materials.

Before deriving the quasi-linear theory, it is useful to introduce some standard definitions and notations. The deformation gradient tensor **F** is defined by

3.1 $$F(s)=\left\{ \begin{array}{ll} I, & s\in (-\infty ,0),\\ \frac{\partial x(s)}{\partial X}, & s\in [0,t], \end{array}\right.$$

with **x**(*s*) denoting the position of a generic particle *P* at time *s* ∈ [0,*t*], and **X** its position at the initial reference time. Note that the start time of the deformation, and any imposed tractions, will be taken as *t*=0. The quantity *J*=det **F**, expressing the local volume change, is a constant *J*=1 when the deformation is isochoric. Furthermore, from the deformation gradient tensor **F** the left Cauchy–Green tensor **B**=**FF**^T^ is obtained, together with its principal invariants

3.2 $$I_{1}=\mathrm{tr}B,\;I_{2}=\frac{1}{2}[{\left(\mathrm{tr}B\right)}^{2}-\mathrm{tr}B^{2}]=(\det B)\mathrm{tr}(B^{-1})\;\mathrm{and}\;I_{3}=\det B=J^{2},$$

which, alternatively, can be expressed in terms of the principal stretches through

3.3 $$I_{1}=\lambda_{1}^{2}+\lambda_{2}^{2}+\lambda_{3}^{2},\;I_{2}=\lambda_{1}^{2}\lambda_{2}^{2}+\lambda_{2}^{2}\lambda_{3}^{2}+\lambda_{1}^{2}\lambda_{3}^{2}\;\mathrm{and}\;I_{3}=\lambda_{1}^{2}\lambda_{2}^{2}\lambda_{3}^{2}.$$

Now, Fung [2] makes the assumption that the QLV stress depends linearly on the (superposed) time history of a related nonlinear elastic response (a nonlinear instantaneous measure of strain). This allows, for example, for incorporation of a finite hyperelastic theory in the analysis. In index notation, Fung's theory (1.1) can be written as^1^

3.4 $$\Pi_{ij}(t)=\int_{-\infty }^{t}G_{ijkl}(t-\tau )\frac{\partial \Pi_{kl}^{e}(\tau )}{\partial \tau }d\tau .$$

Fung refers to *G*_*ijkl*_ as a *reduced relaxation function tensor*. The ‘crucial’ simplification of Fung's theory is that this term is *independent* of the strain. Moreover, if the material is isotropic then **G**, a tensor of rank four, has just two independent components, being therefore consistent with linear theory.^2^

Following the analysis of the previous section, for isotropic materials it is convenient to split the equivalent (instantaneous) *Cauchy stress* into two parts, one which accounts for *microscopic isochoric deformations* of the material and the other that measures purely *compressive deformations*. These two components can be expected to have different instantaneous elastic behaviours as well as distinct relaxation rates, associated for example with the unwinding/unravelling of polymeric fibres as opposed to their stretching. It is assumed that this decomposition can be achieved by taking the deviatoric and hydrostatic components of the equivalent elastic Cauchy stress:

3.5 $$T^{e}=T_{D}^{e}+T_{H}^{e},$$

which can be expressed as

3.6 $$T_{H}^{e}=\frac{1}{3}\mathrm{tr}(T^{e})I\;\mathrm{and}\;T_{D}^{e}=T^{e}-\frac{1}{3}\mathrm{tr}(T^{e})I.$$

The split of equation (3.5) into shear and dilatational components is the nonlinear (hyperelastic) analogue of linear stress–strain law (2.1). However, it cannot be generalized to viscoelasticity, as in (2.2), because it would not preserve *objectivity*. Instead, the second Piola–Kirchhoff stress tensor associated with (3.5) has to be introduced, which is defined by

3.7 $$\Pi^{e}=JF^{-1}T^{e}F^{-T},$$

with

3.8 $$\Pi^{e}=\Pi_{D}^{e}+\Pi_{H}^{e},$$

in which

3.9 $$\Pi_{D}^{e}=JF^{-1}T_{D}^{e}F^{-T}\;\mathrm{and}\;\Pi_{H}^{e}=JF^{-1}T_{H}^{e}F^{-T}.$$

It must be emphasized that the subscripts D and H **do not** refer to the deviatoric and hydrostatic parts of the second Piola–Kirchhoff stress, but correspond to the second Piola–Kirchhoff stress of the deviatoric and hydrostatic Cauchy stress components, respectively. Assuming a superposition principle as for the linear case, it is now possible to introduce an objective viscoelastic law, relating the second Piola–Kirchhoff stress to the past history of the nonlinear rate of strain measure. This is taken as

3.10 $$\Pi (t)=\int_{-\infty }^{t}D(t-s)\frac{\partial }{\partial s}\Pi_{D}^{e}(s)ds+\int_{-\infty }^{t}H(t-s)\frac{\partial }{\partial s}\Pi_{H}^{e}(s)ds,$$

where now D(t) and H(t) are two scalar (independent)-reduced relaxation functions (with D(0)=H(0)=1 without loss of generality). The latter relaxation functions relate to the inherent viscous processes involved with instantaneous shear and compressional (volumetric) deformations, respectively. Clearly, if the material was anisotropic, then a more complex tensorial relaxation function would be required. Furthermore, pre-multiplying by *J*^−1^**F** and post-multiplying by **F**^T^ yield the Cauchy viscoelastic stress

3.11 $$\begin{array}{rl} T(t) & =J^{-1}F(t)\left(\int_{-\infty }^{t}D(t-s)\frac{\partial }{\partial s}\Pi_{D}^{e}(s)ds\right)F^{T}(t)\\ & \;+J^{-1}F(t)\left(\int_{-\infty }^{t}H(t-s)\frac{\partial }{\partial s}\Pi_{H}^{e}(s)ds\right)F^{T}(t), \end{array}$$

and integrating by parts, following (2.7), gives

3.12 $$\begin{array}{rl} T(t) & =J^{-1}F(t)\left(\Pi_{D}^{e}(t)+\int_{0}^{t}D^{\prime }(t-s)\Pi_{D}^{e}(s)ds\right)F^{T}(t)\\ & \;+J^{-1}F(t)\left(\Pi_{H}^{e}(t)+\int_{0}^{t}H^{\prime }(t-s)\Pi_{H}^{e}(s)ds\right)F^{T}(t). \end{array}$$

As mentioned earlier, to allow for large (nonlinear) deformations, a hyperelastic theory can be employed assuming that the measure of the *effective elastic stress* **Π**^e^ is derived from an elastic potential. Let us then specialize equations (3.5)–(3.12), considering the existence of a strain energy function (SEF) *W*, say, which in the isotropic case is dependent on the principal invariants of the deformation *I*_1_,*I*_2_,*I*_3_, or of the principal stretches λ_1_,λ_2_,λ_3_:

3.13 $$W=W(I_{1},I_{2},I_{3})=\tilde{W}(\lambda_{1},\lambda_{2},\lambda_{3}).$$

The general form of elastic Cauchy stress may be written (e.g. [22,23]) as

3.14 $$T^{e}=\beta_{0}I+\beta_{1}B+\beta_{-1}B^{-1},$$

where *β*_*j*_=*β*_*j*_(*I*_1_,*I*_2_,*I*_3_) are the so-called material (or elastic) response functions. In terms of the strain energy function, they are given by

3.15 $$\begin{array}{rl} & \beta_{0}(I_{1},I_{2},I_{3})=\frac{2}{J}[I_{2}W_{2}+I_{3}W_{3}],\\ & \beta_{1}(I_{1},I_{2},I_{3})=\frac{2}{J}W_{1}\\ \text{and}\;\; & \beta_{-1}(I_{1},I_{2},I_{3})=-2JW_{2}, \end{array}\}$$

where

3.16 $$W_{k}=\frac{\partial W}{\partial I_{k}},\;k=1,2,3.$$

It is straightforward to calculate the trace of **T**^e^,

3.17 $$\mathrm{tr}T^{e}=3\beta_{0}+I_{1}\beta_{1}+\frac{I_{2}\beta_{-1}}{I_{3}}$$

and so the deviatoric and hydrostatic elastic Cauchy stress components in (3.6) become

3.18 $$\begin{array}{rl} & T_{D}^{e}=\frac{2}{J}\left[\frac{1}{3}(I_{2}W_{2}-I_{1}W_{1})I+W_{1}B-I_{3}W_{2}B^{-1}\right]\\ \text{and}\;\; & T_{H}^{e}=\frac{2}{J}\left(\frac{2}{3}I_{2}W_{2}+\frac{1}{3}I_{1}W_{1}+I_{3}W_{3}\right)I. \end{array}\}$$

From this, the second Piola–Kirchhoff counterparts, (3.9), are given by

3.19 $$\Pi_{D}^{e}=2\left[\frac{1}{3}(I_{2}W_{2}-I_{1}W_{1})C^{-1}+W_{1}I-I_{3}W_{2}C^{-2}\right]$$

and

3.20 $$\Pi_{H}^{e}=2\left(\frac{2}{3}I_{2}W_{2}+\frac{1}{3}I_{1}W_{1}+I_{3}W_{3}\right)C^{-1},$$

where it is recalled that **C**=**F**^T^**F** is the right Cauchy–Green deformation tensor. The viscoelastic stress is obtained from (3.19) to (3.20) via (3.10) or for the Cauchy stress from (3.12).

Note that (as required) objectivity is now preserved for the viscoelastic model (3.11), but the first part is *not* deviatoric, and so the second term is not purely hydrostatic. In fact, in general both the compressive and shear components of the stress history contribute to the deviatoric and hydrostatic parts of **T**. However, the main point to note is that when there is no deformation, i.e. the principal stretches are unity, λ_*j*_=1,*j*=1,2,3 and **B**≡**I**, then *I*_1_=*I*_2_=3,*I*_3_=1, which reveals that the effective deviatoric elastic stress **T**^e^_D_ vanishes. Similarly, **T**^e^_H_ vanishes as the strain energy function has always to satisfy the additional relation (e.g. [22])

3.21 $$W_{1}(3,3,1)+2W_{2}(3,3,1)+W_{3}(3,3,1)\equiv 0.$$

Thus, both terms **Π**^e^_D_ and **Π**^e^_H_ vanish as $\lambda_{i}\to 1$, and hence, as there is no applied stress until the initial time *t*=0, justifies taking the integration range for the pair of integrals in (3.12) as 0 to *t*.

If it proves convenient to express the strain energy function in terms of the principal stretches $W=\tilde{W}(\lambda_{1},\lambda_{2},\lambda_{3})$, then for diagonal **F** the quasi-linear viscoelastic stress relation (3.12) may be rewritten as

3.22 $$\begin{array}{rl} T_{i}(t) & =J^{-1}\lambda_{i}^{2}(t)\left(\Pi_{Di}^{e}(t)+\int_{0}^{t}D^{\prime }(t-s)\Pi_{Di}^{e}(s)\;ds\right)\\ & \;+J^{-1}\lambda_{i}^{2}(t)\left(\Pi_{Hi}^{e}(t)+\int_{0}^{t}H^{\prime }(t-s)\Pi_{Hi}^{e}(s)\;ds\right), \end{array}$$

in which

3.23 $$\Pi_{Di}^{e}=\frac{{\tilde{W}}_{i}}{\lambda_{i}}-\frac{1}{3\lambda_{i}^{2}}\sum_{j=1}^{3}\lambda_{j}{\tilde{W}}_{j}\;\mathrm{and}\;\Pi_{Hi}^{e}=\frac{1}{3\lambda_{i}^{2}}\sum_{j=1}^{3}\lambda_{j}{\tilde{W}}_{j},$$

where now ${\tilde{W}}_{i}$ refers to the derivative $\partial \tilde{W}/\partial \lambda_{i}$.

The final consideration of this section is the constraint of incompressibility for all possible deformations, i.e. *J*≡1. In this limit, the speed of propagation of compressive disturbances tends to infinity, and hence the relaxation time for viscous dilatational motions can be assumed to tend to zero. Therefore, the second term in (3.11) (or (3.12)), in an analogous fashion to that for linear elasticity (2.7), reduces to

3.24 $$J^{-1}F(t)\left(\Pi_{H}^{e}(t)+\int_{0}^{t}H^{\prime }(t-s)\Pi_{H}^{e}(s)ds\right)F^{T}(t)\to -p(t)I,$$

where *p*(*t*) can be considered as a Lagrange multiplier. Note that this expression in itself is not hydrostatic. It contains both a hydrostatic term, and a component that combines with the first term in (3.12) to make it deviatoric; hence,

3.25 $$T(t)=F(t)\left(\Pi_{D}^{e}(t)+\int_{0}^{t}D^{\prime }(t-s)\Pi_{D}^{e}(s)ds\right)F^{T}(t)-pI,$$

where now from the first equation in (3.19),

3.26 $$\Pi_{D}^{e}(t)=2\left[\left(\frac{I_{2}}{3}W_{2}-\frac{I_{1}}{3}W_{1}\right)C^{-1}+W_{1}I-W_{2}C^{-2}\right].$$

As described above, the present theory has been developed for isotropic materials. The study of *anisotropic* nonlinear viscoelastic materials rapidly becomes rather demanding, although strain measures for anisotropic QLV were discussed in [24]. It is expected that, despite the complexity, the present approach is extendable to certain classes of anisotropy.

## 4. Uniaxial elongation

One of the simplest, useful and hence most popular experiments to measure the properties of a material is to subject a specimen to a simple elongation test. There is a huge literature of results on such a deformation, and it is therefore useful to examine this homogeneous solution, comparing the present result with extant published work. To appreciate the general QLV model developed herein, and the approach needed to obtain a solution, three specific cases will be examined. First, in §4*a* a uniaxial stretch is imposed on the specimen, with the stress determined as a relaxing function of the history of the stretch. For comparison, both Yeoh and Mooney–Rivlin incompressible strain energy functions are considered. In §4*b*, a tensile load is imposed, and the stretch history (creep) determined from this. In this case, the integral equation must be solved numerically, which is the focus of discussion in appendix A. Finally, in §4*c*, the procedure in §4*a* is repeated for a compressible material, with the effect of compressibility on the viscoelastic behaviour highlighted.

### (a) Simple extension

In the case of simple extension, assumed homogeneous (in space) and incompressible, the principal stretches may be specified as

4.1 $$x_{1}(t)=\lambda_{1}(t)X_{1},\;x_{2}(t)=\lambda_{2}(t)X_{2}\;\mathrm{and}\;x_{3}(t)=\lambda_{2}(t)X_{3},$$

where (*X*_1_,*X*_2_,*X*_3_) and (*x*_1_,*x*_2_,*x*_3_) are the Cartesian coordinates in the undeformed and deformed state, respectively, and the stresses are

4.2 $$T_{11}(t)=T(t),\;T_{22}(t)=T_{33}(t)=0\;\mathrm{and}\;T_{ij}=0\;(i\neq j),$$

having assumed that the lateral surfaces are free of stress. The deformation gradient *F*_*ij*_=∂*x*_*i*_/∂*X*_*j*_ is of diagonal form

4.3 $$F(t)=\mathrm{diag}(\lambda_{1}(t),\lambda_{2}(t),\lambda_{2}(t))$$

and the constraint of incompressibility, *J*=1, gives a relationship between the stretches λ_1_ and λ_2_, in particular $\lambda_{2}=\lambda_{1}^{-1/2}$. Setting λ_1_=λ, the deformation gradient tensor **F** and the left Cauchy–Green tensor **B**=**FF**^T^ become

4.4 $$F(t)=\mathrm{diag}(\lambda (t),\lambda^{-1/2}(t),\lambda^{-1/2}(t))\;\mathrm{and}\;B(t)=\mathrm{diag}(\lambda^{2}(t),\lambda^{-1}(t),\lambda^{-1}(t)).$$

The principal invariants are therefore

4.5 $$I_{1}=\lambda^{2}+\frac{2}{\lambda },\;I_{2}=2\lambda +\frac{1}{\lambda^{2}}\;\mathrm{and}\;I_{3}=1,$$

and hence taking the diagonal terms of equation (3.25) yields the principal Cauchy stresses

4.6 $$T_{11}(t)=T(t)=\lambda^{2}(t)\left(\Pi_{D11}^{e}(t)+\int_{0}^{t}D^{\prime }(t-s)\Pi_{D11}^{e}(s)\;ds\right)-p$$

and

4.7 $$T_{22}(t)=T_{33}(t)=0=\lambda^{-1}(t)\left(\Pi_{D22}^{e}(t)+\int_{0}^{t}D^{\prime }(t-s)\Pi_{D22}^{e}(s)\;ds\right)-p,$$

respectively. Thus, by subtraction, the Lagrange multiplier *p* can be eliminated, and so it is possible to rewrite the stress–strain relationship (4.6) and (4.7) as

4.8 $$\begin{array}{rl} T(t) & =\lambda^{2}(t)\Pi_{D11}^{e}(t)-\frac{1}{\lambda (t)}\Pi_{D22}^{e}(t)\\ & \;+\int_{0}^{t}D^{\prime }(t-s)\left(\lambda^{2}(t)\Pi_{D11}^{e}(s)-\frac{1}{\lambda (t)}\Pi_{D22}^{e}(s)\right)\;ds, \end{array}$$

where from (3.26)

4.9 $$\Pi_{D11}^{e}=2\left[\frac{2}{3}\left(W_{1}+\frac{W_{2}}{\lambda }\right)\left(1-\frac{1}{\lambda^{3}}\right)\right]$$

and

4.10 $$\Pi_{D22}^{e}=2\left[\frac{1}{3}\left(W_{1}+\frac{W_{2}}{\lambda }\right)(1-\lambda^{3})\right].$$

Note that equations (4.9) and (4.10) depend on the specific choice of strain energy function *W*, and so it is useful to examine two specific examples. Let us start by assuming the instantaneous response is modelled by a two-term Yeoh strain energy function [25]

4.11 $$W=\frac{\mu }{4}(2(I_{1}-3)+\alpha {\left(I_{1}-3\right)}^{2}),$$

which yields the stress–strain relationship

4.12 $$T=-pI+\mu (1-3\alpha +\alpha I_{1})B,$$

where *α* is a positive constant and *μ* is the shear modulus from infinitesimal theory. Thus,

4.13 $$2W_{1}=\mu (1-3\alpha +\alpha I_{1}),\;W_{2}=0.$$

It is straightforward to obtain from (4.8) the relation

4.14 $$\begin{array}{rl} \frac{T(t)}{\mu } & =k(t)\left(\lambda (t)-\frac{1}{\lambda^{2}(t)}\right)+\int_{0}^{t}D^{\prime }(t-s)k(s)\\ & \;\times \left(\lambda^{2}(t)\left[\frac{2}{3}\left(\frac{1}{\lambda (s)}-\frac{1}{\lambda^{4}(s)}\right)\right]-\frac{1}{\lambda (t)}\left[\frac{1}{3}\left(\frac{1}{\lambda (s)}-\lambda^{2}(s)\right)\right]\right)\;ds, \end{array}$$

where

4.15 $$k(t)=2\alpha +(1-3\alpha )\lambda (t)+\alpha \lambda^{3}(t).$$

The second example is the instantaneous response modelled by a Mooney–Rivlin strain energy function

4.16 $$W=\frac{\mu }{2}\left(\frac{1}{2}+\gamma \right)(I_{1}-3)+\frac{\mu }{2}\left(\frac{1}{2}-\gamma \right)(I_{2}-3),$$

which yields the stress–strain relationship

4.17 $$T=-pI+\mu \left[\left(\frac{1}{2}+\gamma \right)B-\left(\frac{1}{2}-\gamma \right)B^{-1}\right],$$

where *γ* is a constant in the range −1/2≤*γ*≤1/2. Note that (4.17) reduces to the neo-Hookean model when *γ*=1/2. The partial derivatives of *W* are

4.18 $$2W_{1}=\mu \left(\frac{1}{2}+\gamma \right)\;\mathrm{and}\;2W_{2}=\mu \left(\frac{1}{2}-\gamma \right),$$

and so (4.8) becomes, after simplification,

4.19 $$\begin{array}{rl} \frac{T(t)}{\mu } & =\frac{1}{2}\ell (s)\left(\lambda (t)-\frac{1}{\lambda^{2}(t)}\right)+\frac{1}{6}\int_{0}^{t}D^{\prime }(t-s)\ell (s)\\ & \;\times \left[2\lambda^{2}(t)\left(\frac{1}{\lambda (s)}-\frac{1}{\lambda^{4}(s)}\right)+\frac{1}{\lambda (t)}\left(\lambda^{2}(s)-\frac{1}{\lambda (s)}\right)\right]\;ds, \end{array}$$

in which

4.20 ℓ(t)=[1−2γ+λ(t)(1+2γ)].

These two viscoelastic models can be compared by considering an ‘experiment’ where the stretch is imposed and the stress is measured. To specify matters, the stress relaxation function is chosen to be the classical one-term Prony series

4.21 $$D(t)=\frac{\mu_{\infty }}{\mu }+\left(1-\frac{\mu_{\infty }}{\mu }\right)e^{-t/\tau },$$

where *μ*, $\mu_{\infty }$ are the infinitesimal shear modulus and the long-time infinitesimal shear modulus, respectively, and *τ* is the relaxation time. These are set as

4.22 $$\frac{\mu_{\infty }}{\mu }=0.5\;\mathrm{and}\;\tau =1.0s.$$

**Figure 1.**
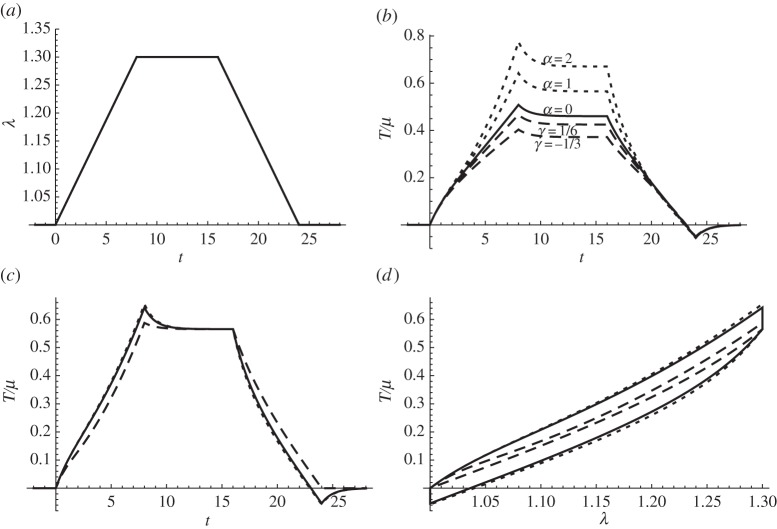
The imposed stretch history is shown in graph (*a*). The resultant dimensionless stress *T*/*μ* is plotted in graph (*b*), found for the Yeoh model (4.14) (dotted) and for the Mooney–Rivlin material (4.19) (dashed) where the solid curve is the neo-Hookean limit *α*=0 (or *γ*=1/2). (*c*) *T*/*μ* is plotted from the predictions of (4.24) (dotted), (4.23) (dashed) and (4.14) (solid), respectively. (*d*) The dimensionless stress, *T*/*μ*, is plotted against stretch, λ, from the predictions of (4.24) (dotted), (4.23) (dashed) and (4.14) (solid).

A dynamic imposed stretch history (shown in figure 1*a*) is applied. The time variation is assumed slow so that inertial terms in the balance equations can be neglected. Figure 1*b* shows the resultant stress predictions *T*/*μ* for the Yeoh hyperelastic model (4.14) (dotted curves with *α*=1,2) and for the Mooney–Rivlin hyperelastic model (4.19) (dashed curves with *γ*=1/6,−1/3). It is interesting to observe that the two hyperelastic models depart from the solid curve, obtained when the strain energy function is of the neo-Hookean type, i.e. *α*=0,*γ*=1/2, in different ways. The Yeoh material is found to harden as the parameter *α* increases, whereas the effect of decreasing *γ* from 1/2 leads to a softening of the Mooney–Rivlin material's behaviour.

As mentioned in the Introduction, it is useful to contrast the results presented here with those discussed, for example, by Ciambella *et al.* [15]. In that article, the authors employed a form of QLV, which, with the incompressible Yeoh SEF, yields the stretch–stress law (see eqn (16) in [15]):

4.23 $$\begin{array}{rl} \frac{T(t)}{\mu } & =[\lambda (t)-\lambda^{-2}(t)][2\alpha +(1-3\alpha )\lambda (t)+\alpha \lambda^{3}(t)]\\ & \;+[\lambda^{2}(t)-\lambda^{-1}(t)]\int_{0}^{t}D^{\prime }(t-s)\lambda^{-1}(s)[2\alpha +(1-3\alpha )\lambda (s)+\alpha \lambda^{3}(s)]\;ds. \end{array}$$

Similar equations can be derived from the constitutive models in [16] or [17]. In Ciambella *et al.*'s article, their equation (4.23) was compared with that offered by the formulation used in the ABAQUS finite-element analysis (FEA) package (Version 6.7, see [26]), namely

4.24 $$\begin{array}{rl} \frac{T(t)}{\mu } & =[\lambda (t)-\lambda^{-2}(t)][2\alpha +(1-3\alpha )\lambda (t)+\alpha \lambda^{3}(t)]\\ & \;+\int_{0}^{t}D^{\prime }(t-s)[\lambda (s)-\lambda^{-2}(s)][2\alpha +(1-3\alpha )\lambda (s)+\alpha \lambda^{3}(s)]\;ds. \end{array}$$

The three alternative viscoelastic expressions (4.24), (4.23) and the present result (4.14) may be conveniently compared by again imposing a stretch and determining the resultant dimensionless stress *T*/*μ*. Employing the same stretch history as above (figure 1*a*), the relaxation function in (4.21) with parameters as in (4.22) and *α*=1.0, yields the curves in figure 1*c* predicted from (4.24) (ABAQUS: dotted), (4.23) (Ciambella *et al.*: dashed) and (4.14) (De Pascalis *et al.*: solid), respectively. An alternative way of representing this deformation is via a stretch–stress diagram (figure 1*d*). It is clear that the present QLV approach, illustrated by the solid curve in both figures, gives a result that lies remarkably close to that found from the ABAQUS model, whereas Ciambella *et al.*'s solution is quite distinct. The latter model was developed by the authors in order to address the deficiency they highlighted regarding v. 6.7 of ABAQUS. However, their result also exhibits a substantial limitation: it predicts instantaneous zero stress whenever λ recovers back to unity after some (arbitrary) deformation, thus showing no ‘memory’ of the history of the stress in this situation. This is at variance with physically observed behaviour, and in particular that found for linear viscoelasticity.

### (b) Simple tensile load

Rather than an imposed stretch, perhaps a more important experiment for measuring viscoelastic properties of materials is application of a simple tensile load: a slowly varying uniaxial stress is imposed on the body for which the resultant stretch is measured over time. For linear viscoelasticity, equation (2.2) can be inverted to yield a straightforward creep relation to determine the strain. However, for nonlinear viscoelasticity, the solution procedure is somewhat more difficult, as inversion is not possible. A typical QLV relation is given in (4.19), which may be considered in the general form

4.25 $$T(t)=g(\lambda (t))+\sum_{j=1}^{N}f_{j}(\lambda (t))\int_{0}^{t}D^{\prime }(t-s)h_{j}(\lambda (s))\;ds.$$

Although the integrand is of separable type, the presence of the various *f*_*j*_(λ(*t*)) terms means that an inversion operator cannot be introduced. Instead, a numerical scheme must be employed that can evaluate the stretch as a function of time, subjected to a prescribed stress. A suitable numerical discretization procedure is offered in appendix A, which has error *O*(*δt*^4^), where *δt* is the step size and so gives rapid convergence.

**Figure 2.**
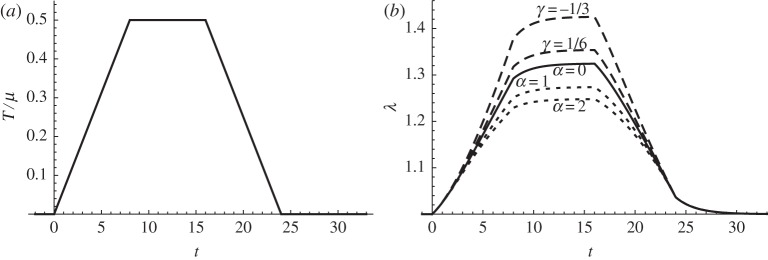
Plot of the dimensionless stress history *T*/*μ* (*a*) and the resultant stretch λ (*b*), from the Yeoh model predictions (4.14) (dotted) and that from Mooney–Rivlin predictions (4.19) (dashed). The solid curve is the neo-Hookean limit *α*=0 (or *γ*=1/2).

An example is illustrated in figure 2, with the imposed stress history shown on the left graph. The one-term Prony series relaxation function (4.21), with constant values as given in equation (4.22), is again employed. The resultant stretch is given in figure 2*b* for the Yeoh strain energy function (4.14) (dotted curves with *α*=1,2), and for the Mooney–Rivlin strain energy function (dashed curves with *α*=1,2). As before, the solid curve is the neo-Hookean prediction (*α*=0,*γ*=1/2) and it can be seen that increasing *α* in the Yeoh model leads to material hardening, while decreasing *γ* leads to softening of the Mooney–Rivlin material.

### (c) Simple extension of a compressible bar

The purpose of this section is to repeat that analysis in §4*a* for a compressible material. As before, a bar, with zero tractions on its lateral faces, is assumed to undergo simple homogeneous extension, caused by an imposed stress or stretch in the *X*_1_ direction, say. Then, the principal stretches may be given by

4.26 $$x_{1}(t)=\lambda_{1}(t)X_{1},\;x_{2}(t)=\lambda_{2}(t)X_{2}\;\mathrm{and}\;x_{3}(t)=\lambda_{2}(t)X_{3},$$

where symmetry between the *X*_2_ and *X*_3_ directions is clear, and so the deformation gradient tensor is

4.27 $$F(t)=\text{diag}(\lambda_{1}(t),\lambda_{2}(t),\lambda_{2}(t))$$

and the left Cauchy–Green tensor **B** becomes

4.28 $$B(t)=\text{diag}(\lambda_{1}^{2}(t),\lambda_{2}^{2}(t),\lambda_{2}^{2}(t)).$$

The principal invariants are therefore

4.29 $$I_{1}(t)=\lambda_{1}^{2}(t)+2\lambda_{2}^{2}(t),\;I_{2}(t)=2\lambda_{1}^{2}(t)\lambda_{2}^{2}(t)+\lambda_{2}^{4}(t)\;\mathrm{and}\;I_{3}=\lambda_{1}^{2}(t)\lambda_{2}^{4}(t).$$

The above can be substituted into the governing viscoelastic equation (3.12), with effective elastic stresses (3.19) and (3.20), and simplified. The *X*_1_ component of the stress yields, after simplification,

4.30 T(t)=λ1(t)λ22(t)[43∫0tD′(t−s)(1−λ22(s)λ12(s))(W1(s)+W2(s)λ22(s)) ds+23∫0tH′(t−s){W1(s)+2(W1(s)λ12(s)+2W2(s))λ22(s)+(2W2(s)λ12(s)+3W3(s))λ24(s)} ds+2(W1(t)+2W2(t)λ22(t)+W3(t)λ24(t))(1−λ22(s)λ12(s))],

and the normal stresses in the *X*_2_ and *X*_3_ equations are both

4.31 0=1λ1(t)[23∫0tD′(t−s)(1−λ12(s)λ22(s))(W1(s)+W2(s)λ22(s)) ds+23∫0tH′(t−s)(W1(s)λ12(s)λ22(s)+2(W1(s)+2W2(s)λ12(s))+(2W2(s)+3W3(s)λ12(s))λ22(s)λ12(s)λ22(s)) ds+2(W1(t)+W2(t)λ12(t)+(W2(t)+W3(t)λ12(t))λ22(t))(1−λ12(s)λ22(s))],

where as before *W*_*j*_ is the derivative of *W* with respect to the indicated invariant (3.16). Clearly, the particular choice of the strain energy function affects the results and so, as before, a couple of examples are presented. Assuming the Horgan–Murphy strain energy function [27], used for modelling a material with a small compressibility, the SEF is

4.32 $$W=\frac{\mu }{2}\left(\frac{1}{2}+\gamma \right)(I_{1}-3I_{3}^{1/3})+\frac{\mu }{2}\left(\frac{1}{2}-\gamma \right)(I_{2}-3I_{3}^{2/3})+\frac{\kappa }{2}{\left(I_{3}^{1/2}-1\right)}^{2},$$

where *γ* is an arbitrary constant, *μ* is the infinitesimal shear modulus and *κ* is the infinitesimal bulk modulus. Then

$$W_{1}=\frac{\mu }{2}\left(\frac{1}{2}+\gamma \right),\;W_{2}=\frac{\mu }{2}\left(\frac{1}{2}-\gamma \right)$$

and

$$W_{3}=-\frac{\mu }{2}\left(\frac{1}{2}+\gamma \right)I_{3}^{-2/3}-\mu \left(\frac{1}{2}-\gamma \right)I_{3}^{-1/3}+\frac{\kappa }{2}(1-I_{3}^{-1/2}),$$

which are substituted in (4.30) and (4.31). Setting *γ*=1/2, for ease of presentation, the stresses become

4.33 T(t)=1λ1(t)λ22(t)[λ12(t)(μ+2μ3∫0tD′(t−s)(1−λ22(s)λ12(s)) ds+13∫0tH′(t−s)(μ(2λ22(s)λ12(s)+1−3λ24/3(s)λ14/3(s))+3κ(λ24(s)−λ22(s)λ1(s))) ds+κλ24(t))−λ12/3(t)λ24/3(t)(μ+κλ11/3(t)λ22/3(t))43]

and

4.34 0=1λ1(t)λ22(t)[λ22(t)(μ+μ3∫0tD′(t−s)(1−λ12(s)λ22(s)) ds+13∫0tH′(t−s)(μ(2+λ12(s)λ22(s)−3λ12/3(s)λ22/3(s))+3κλ1(s)(λ1(s)λ22(s)−1)) ds)+κλ12(t)λ24(t)−λ12/3(t)λ24/3(t)(μ+κλ11/3(t)λ22/3(t))43],

respectively. By contrast, for the Gent model (e.g. [28]),

$$W=\frac{\mu }{2}J_{m}\log{\left(1-\frac{I_{1}-3}{J_{m}}\right)}^{-1}+\frac{\kappa }{2}{\left(\frac{I_{3}-1}{2}-\frac{1}{2}\log I_{3}\right)}^{4},$$

where *J*_*m*_ is a constant limiting value for *I*_1_−3, taking into account the finite extensibility of polymeric chains within the material. Hence,

4.35 $$W_{1}=\frac{\mu }{2}{\left(1-\frac{I_{1}-3}{J_{m}}\right)}^{-1},\;W_{2}=0\;\mathrm{and}\;W_{3}=\frac{\kappa }{8}\left(1-\frac{1}{I_{3}}\right){\left(I_{3}-1-\log I_{3}\right)}^{3},$$

which can then substituted into (4.30) and (4.31) to yield the equivalent results for the stresses. These are omitted for brevity.

## 5. Concluding remarks

This paper has focused on reappraising Fung's method for QLV. It has been shown that some of the negative features commonly associated with the approach are merely a consequence of the way it has been applied elsewhere. The approach outlined herein was shown in §4 to yield ‘sensible’ results and offers a straightforward approach in solving a wide range of models. The present method exhibits similarities with Simo's approach to nonlinear viscoelasticity [7], although the latter assumed a scalar relaxation function acting on the stress. Therefore, it is noted that Simo's method would not reduce in the linear limit to the usual relation (2.3) if the latter shear and bulk moduli have different temporal behaviour. (Note that ABAQUS v. 6.12 employs the same relaxation time constants in both the shear and bulk parts of the field.) Herein, the relaxation function is a tensor, which for isotropic materials reduces to two distinct scalar relaxation functions, one acting on the compressive part and the other on the shear component of the stress. This therefore is consistent with that found for linear theory.

It was shown that for an imposed deformation (e.g. stretch) it is simple matter to solve the QLV equation directly. For imposed stress, the stretch has to be deduced by solving (inverting) the integral equation, and this is achieved here using a discretized scheme accurate to an order of the cube of the time-step size. Muliana *et al.* [29] have recently offered a QLV model where the strain is expressed as a function of the stress, which may be viewed as a dual model to (1.1). However, it is not clear as yet whether their approach is as effective at modelling viscoelasticity as Fung's scheme.

The authors are currently applying the present approach to a range of problems in viscoelasticity. The numerical procedure described in appendix A can be employed in a straightforward manner for any deformation that is incompressible and homogeneous, for example simple shear. It is presently being extended so that it can used for compressible materials undergoing simple extension or shear. The QLV method is also adaptable to inhomogeneous problems, for example those studied in [30], large amplitude radial deformations in two and three dimensions, and simple torsion. Such models lead to partial differential nonlinear Volterra integral equations and extending the present numerical scheme to these should offer improvement over current methods. It is also anticipated that the present approach will be more suited to satisfying the equations of equilibrium than, say, the more heuristic QLV relation employed in ABAQUS (v. 6.12). Finally, the authors are using the model for several biomechanics problems, to derive a perturbation theory for the viscoelastic evolution of small deformations on the top of large ones and to derive effective properties of ligaments and other tissues.

## Footnotes

1 Note, in order to clarify exposition, the space variable in (1.1) is omitted; the same will be done henceforth, as long as no confusion occurs.

2 This is not always taken in account, for example in [1,21] the authors introduce an isotropic model which contains only one scalar relaxation function *G*.

## Appendix A. A numerical high-order solution procedure for Volterra integral equations

The purpose of this appendix is to offer a simple and rapid numerical scheme for solving Volterra integral equations of the type obtained by the QLV approach (see e.g. (4.14)). It is assumed that they have the separable form ,

A 1 $$T(t)=g(\lambda (t))+\sum_{j=1}^{N}f_{j}(\lambda (t))\int_{0}^{t}D^{\prime }(t-s)h_{j}(\lambda (s))\;ds,$$

where $T(t),D(t),g(X),f_{j}(X)$ and *h*_*j*_(*X*) are all known functions of their respective arguments. Clearly, all the equations offered in §4*a* lie within this class but for more general problems, with inhomogeneous deformations, the analysis described below must be amended.

Let us now discretize the system and evaluate the equation at each time *t*_*n*_=*nδt*, with *n*=0,1,2,3,…,*n*_max_, where *δt* is a *small* time step. On writing the stretch at each time step as

A 2 $$\lambda_{n}=\lambda (t_{n})=\lambda (n\delta t),$$

a Taylor series expansion can be employed to yield

A 3 $$\begin{array}{rl} g(\lambda_{n}) & =g(\lambda_{n-1})+g^{\prime }(\lambda_{n-1})(\lambda_{n}-\lambda_{n-1})+\frac{g^{\prime \prime }(\lambda_{n-1})}{2!}{\left(\lambda_{n}-\lambda_{n-1}\right)}^{2}\\ & \;+\frac{g^{\prime \prime \prime }(\lambda_{n-1})}{3!}{\left(\lambda_{n}-\lambda_{n-1}\right)}^{3}+O{\left((\lambda_{n}-\lambda_{n-1}\right)}^{4}), \end{array}$$

where ′ denotes differentiation with respect to the argument, and

A 4 $$\lambda_{n}=\lambda (n\delta t)=\lambda_{n-1}+\lambda_{n-1}^{\prime }\delta t+\frac{\lambda_{n-1}^{\prime \prime }}{2!}\delta t^{2}+\frac{\lambda_{n-1}^{\prime \prime \prime }}{3!}\delta t^{3}+O(\delta t^{4}).$$

Note that all functions are assumed sufficiently smooth between adjacent time steps so that the relations above and below hold. From (A 4), it is clear that

A 5 $$\begin{array}{rl} & (\lambda_{n}-\lambda_{n-1})=\lambda_{n-1}^{\prime }\delta t+\frac{\lambda_{n-1}^{\prime \prime }}{2!}\delta t^{2}+\frac{\lambda_{n-1}^{\prime \prime \prime }}{3!}\delta t^{3}+O(\delta t^{4}),\\ & {\left(\lambda_{n}-\lambda_{n-1}\right)}^{2}={\left(\lambda_{n-1}^{\prime }\right)}^{2}\delta t^{2}+\lambda_{n-1}^{\prime }\lambda_{n-1}^{\prime \prime }\delta t^{3}+O(\delta t^{4})\\ \text{ and }\;\; & {\left(\lambda_{n}-\lambda_{n-1}\right)}^{3}={\left(\lambda_{n-1}^{\prime }\right)}^{3}\delta t^{3}+O(\delta t^{4}). \end{array}\}$$

The integral in (A 1) can also be expanded as

A 6 $$\begin{array}{rl} \int_{0}^{t}D^{\prime }(t-s)h_{j}(\lambda (s))\;ds & =\int_{0}^{n\delta t}D^{\prime }(n\delta t-s)h_{j}(\lambda (s))\;ds\\ & =\sum_{m=0}^{n-1}\int_{m\delta t}^{(m+1)\delta t}D^{\prime }(n\delta t-s)h_{j}(\lambda (s))\;ds\\ & =\sum_{m=0}^{n-1}\int_{0}^{\delta t}D^{\prime }((n-m)\delta t-u)h_{j}(\lambda (m\delta t+u))du, \end{array}$$

where in the latter the substitution *s*=*mδt*+*u* is used. The integrand functions can be further expanded as

A 7 $$\begin{array}{rl} D^{\prime }((n-m)\delta t-u) & =D^{\prime }((n-m)\delta t)-D^{\prime \prime }((n-m)\delta t)u\\ & \;+\frac{D^{\prime \prime \prime }((n-m)\delta t)}{2!}u^{2}+O(u^{3}) \end{array}$$

and

A 8 $$\begin{array}{rl} h_{j}(\lambda (m\delta t+u)) & =h_{j}(\lambda_{m})+\frac{dh_{j}(\lambda_{m})}{du}u+\frac{1}{2!}\frac{d^{2}h_{j}(\lambda_{m})}{du^{2}}u^{2}+O(u^{3})\\ & =h_{j}(\lambda_{m})+\lambda_{m}^{\prime }h_{j}^{\prime }(\lambda_{m})u+\frac{1}{2!}(\lambda_{m}^{\prime \prime }h_{j}^{\prime }(\lambda_{m})+{\left(\lambda_{m}^{\prime }\right)}^{2}h_{j}^{\prime \prime }(\lambda_{m}))u^{2}+O(u^{3}), \end{array}$$

and therefore, after some simple algebra and integration, it is found that

A 9 $$\begin{array}{rl} & \int_{0}^{\delta t}D^{\prime }((n-m)\delta t-u)h_{j}(\lambda (m\delta t+u))du=D^{\prime }((n-m)\delta t)h_{j}(\lambda_{m})\delta t\\ & \;+(D^{\prime }((n-m)\delta t)\lambda_{m}^{\prime }h_{j}^{\prime }(\lambda_{m})-D^{\prime \prime }((n-m)\delta t)h_{j}(\lambda_{m}))\frac{\delta t^{2}}{2}\\ & \;+(D^{\prime \prime \prime }((n-m)\delta t)h_{j}(\lambda_{m})-2D^{\prime \prime }((n-m)\delta t)\lambda_{m}^{\prime }h_{j}^{\prime }(\lambda_{m})\\ & \;+D^{\prime }((n-m)\delta t)(\lambda_{m}^{\prime \prime }h_{j}^{\prime }(\lambda_{m})+{\left(\lambda_{m}^{\prime }\right)}^{2}h_{j}^{\prime \prime }(\lambda_{m})))\frac{\delta t^{3}}{6}+O(\delta t^{4}). \end{array}$$

Using now the expansion given in (A 3), with the help of relation (A 5), it is possible to rewrite the expansion for both *g* and *f*_*j*_ as

$$\begin{array}{rl} g(\lambda_{n}) & =g(\lambda_{n-1})+\lambda_{n-1}^{\prime }g^{\prime }(\lambda_{n-1})\delta t+(\lambda_{n-1}^{\prime \prime }g^{\prime }(\lambda_{n-1})+{\left(\lambda_{n-1}^{\prime }\right)}^{2}g^{\prime \prime }(\lambda_{n-1}))\frac{\delta t^{2}}{2}\\ & \;+(\lambda_{n-1}^{\prime \prime \prime }g^{\prime }(\lambda_{n-1})+3\lambda_{n-1}^{\prime }\lambda_{n-1}^{\prime \prime }g^{\prime \prime }(\lambda_{n-1})+{\left(\lambda_{n-1}^{\prime }\right)}^{3}g^{\prime \prime \prime }(\lambda_{n-1}))\frac{\delta t^{3}}{6}+O(\delta t^{4}) \end{array}$$

and

$$\begin{array}{rl} f_{j}(\lambda_{n}) & =f_{j}(\lambda_{n-1})+\lambda_{n-1}^{\prime }f_{j}^{\prime }(\lambda_{n-1})\delta t+(\lambda_{n-1}^{\prime \prime }f_{j}^{\prime }(\lambda_{n-1})+{\left(\lambda_{n-1}^{\prime }\right)}^{2}f_{j}^{\prime \prime }(\lambda_{n-1}))\frac{\delta t^{2}}{2}\\ & \;+(\lambda_{n-1}^{\prime \prime \prime }f_{j}^{\prime }(\lambda_{n-1})+3\lambda_{n-1}^{\prime }\lambda_{n-1}^{\prime \prime }f_{j}^{\prime \prime }(\lambda_{n-1})+{\left(\lambda_{n-1}^{\prime }\right)}^{3}f_{j}^{\prime \prime \prime }(\lambda_{n-1}))\frac{\delta t^{3}}{6}+O(\delta t^{4}). \end{array}$$

Finally, gathering all terms, whole equation (A 1) can be written as the following expansion:

A 10 $$\begin{array}{rl} T(n\delta t) & =g(\lambda_{n-1})+\lambda_{n-1}^{\prime }g^{\prime }(\lambda_{n-1})\delta t+(\lambda_{n-1}^{\prime \prime }g^{\prime }(\lambda_{n-1})+{\left(\lambda_{n-1}^{\prime }\right)}^{2}g^{\prime \prime }(\lambda_{n-1}))\frac{\delta t^{2}}{2}\\ & \;+(\lambda_{n-1}^{\prime \prime \prime }g^{\prime }(\lambda_{n-1})+3\lambda_{n-1}^{\prime }\lambda_{n-1}^{\prime \prime }g^{\prime \prime }(\lambda_{n-1})+{\left(\lambda_{n-1}^{\prime }\right)}^{3}g^{\prime \prime \prime }(\lambda_{n-1}))\frac{\delta t^{3}}{6}\\ & \;+A_{n-1}\delta t+B_{n-1}\frac{\delta t^{2}}{2}+C_{n-1}\frac{\delta t^{3}}{6}+O(\delta t^{4}), \end{array}$$

where *A*_*n*−1_,*B*_*n*−1_,*C*_*n*−1_ are the following expressions:

A 11 $$\begin{array}{rl} A_{n-1} & =\sum_{j=1}^{N}\sum_{m=0}^{n-1}[f_{j}(\lambda_{n-1})D^{\prime }((n-m)\delta t)h_{j}(\lambda_{m})], \end{array}$$

A 12 $$\begin{array}{rl} B_{n-1} & =\sum_{j=1}^{N}\sum_{m=0}^{n-1}[2\lambda_{n-1}^{\prime }f_{j}^{\prime }(\lambda_{n-1})D^{\prime }((n-m)\delta t)h_{j}(\lambda_{m})\\ & \;+f_{j}(\lambda_{n-1})(D^{\prime }((n-m)\delta t)\lambda_{m}^{\prime }h_{j}^{\prime }(\lambda_{m})-D^{\prime \prime }((n-m)\delta t)h_{j}(\lambda_{m}))] \end{array}$$

and

A 13 $$\begin{array}{rl} C_{n-1}=\sum_{j=1}^{N}\sum_{m=0}^{n-1} & \left[f_{j}(\lambda_{n-1})(D^{\prime \prime \prime }((n-m)\delta t)h_{j}(\lambda_{m})-2G^{\prime \prime }((n-m)\delta t)\lambda_{m}^{\prime }h_{j}^{\prime }(\lambda_{m}\right)\\ & \;+D^{\prime }((n-m)\delta t)(\lambda_{m}^{\prime \prime }h_{j}^{\prime }(\lambda_{m})+{\left(\lambda_{m}^{\prime }\right)}^{2}h_{j}^{\prime \prime }(\lambda_{m})))\\ & \;+3\lambda_{n-1}^{\prime }f_{j}^{\prime }(\lambda_{n-1})(D^{\prime }((n-m)\delta t)\lambda_{m}^{\prime }h_{j}^{\prime }(\lambda_{m})-D^{\prime \prime }((n-m)\delta t)h_{j}(\lambda_{m}))\\ & \;+3D^{\prime }((n-m)\delta t)h_{j}(\lambda_{m})(\lambda_{n-1}^{\prime \prime }f_{j}^{\prime }(\lambda_{n-1})+{\left(\lambda_{n-1}^{\prime }\right)}^{2}f_{j}^{\prime \prime }(\lambda_{n-1}))]. \end{array}$$

This equation must hold for each *n* and at each level of approximation. Hence, keeping terms up to *O*(*δt*) in (A 10), and rearranging, gives

A 14 $$\lambda_{n-1}^{\prime }=\frac{\left(T(n\delta t)-g(\lambda_{n-1})-A_{n-1}\delta t\right)}{\left(\delta tg^{\prime }(\lambda_{n-1})\right)}$$

and then equating terms at higher order (*O*(*δt*^2^) and *O*(*δt*^3^)) to zero yields, respectively,

A 15 $$\lambda_{n-1}^{\prime \prime }=-\frac{\left({\left(\lambda_{n-1}^{\prime }\right)}^{2}g^{\prime \prime }(\lambda_{n-1})+B_{n-1}\right)}{g^{\prime }(\lambda_{n-1})}$$

and

A 16 $$\lambda_{n-1}^{\prime \prime \prime }=-\frac{\left(3\lambda_{n-1}^{\prime }\lambda_{n-1}^{\prime \prime }g^{\prime \prime }(\lambda_{n-1})+{\left(\lambda_{n-1}^{\prime }\right)}^{3}g^{\prime \prime \prime }(\lambda_{n-1})+C_{n-1}\right)}{g^{\prime }(\lambda_{n-1})}.$$

Now, the procedure for solving the discretized equations (A 10), for specified stress, is clear. Deformation commences at *t*=0 (or *n*=0) and so from (A 1):

A 17 T(0)=g(λ(0))

gives

A 18 $$\lambda (0)=\lambda_{0}=g^{-1}(T(0)).$$

As there is no stretch prior to an applied stress, an initial zero stress, *T*(0)=0, would mean that λ_0_=1. The derivatives of λ_0_ are derived from (A 14) to (A 16) (setting *n*=1 in these equations) and hence λ_1_ is found from (A 4). Then *n* is incremented, the values of λ′,λ′′,λ′′′ determined, and these are again used to determine the stretch at the next time step. The process is repeated at each following step. Note that size of the sums in *A*_*n*−1_,*B*_*n*−1_,*C*_*n*−1_ grows with *n*, and so for large times, or using small time steps, evaluation of the system can become rather slow. However, this difficulty can be alleviated, with the present system, by obtaining a relationship between *A*_*n*_ and its previous increment *A*_*n*−1_, and similarly for *B*_*n*_,*C*_*n*_. The precise forms of these relationships are determined from the given *f*_*j*_(λ) and the relaxation function D(t). In practice, the solution procedure works well, with an error of *O*(*δt*^4^), i.e. for a time step of *δt*=0.01 then the error is *O*(10^−8^).
